# Medical News and Bulletin

**Published:** 1843-10-28

**Authors:** 


### MEDICAL NEWS AND BULLETIN.

### THE JESUITS TURNED DOCTORS.

The Jesuits are not only aspiring to the monopoly of teaching in France, but also of medicine. The Jesuits of Avignon have circulated throughout that town, and its vicinity, a *brochure* entitled *Notre Dame de 'Remède, ou Médecine Miraculeuse*, which in gross absurdity surpasses any thing yet published of this kind. According to this book, paralysis, dropsy, cancer, blindness, luxations, and all the graver class of affections, are immediately and radically cured by Novenas [nine days prayer to the Virgin or some Saint,] fasts, severe discipline, vows of embracing a religious life, and above all the wearing of medals.

### CLINIC FOR THE DISEASES OF CHILDREN IN LONDON.

Preparations are being made at Guy's Hospital to establish a clinical ward for the diseases of children, the first of the kind in England, to be placed under the superintendence of Dr. Golding Bird.

### PROFESSOR CARR'S INTRODUCTORY LECTURE.

We have recently received a spirited lecture delivered at the opening of the Castleton Medical School, for the autumnal session of 1843, by the Professor of Chemistry, Dr. Ezra S. Carr.

### DEATH OF DR. CHERVIN.

The death of this conscientious enthusiast occurred on the 14th of August last, at Bourbonne-les-Bains, in the Pyrennees, whither he had gone for his health. His whole fortune and the latter portion of life were devoted to the investigation of yellow fever, and the furtility of quarantine regulations. He died miserably poor.

### HOMEOPATHY.

About the time Hahneman departed this life in Paris, the homeopathic institution at Leipsig fell to the ground for want of funds.

### DR. WILLIAMS' PRINCIPLES OF MEDICINE.

An excellent work on Pathology and Therapeutics bearing this name, has been lately issued in London, by the well-known Dr. Williams, of University College.

### DR. WATSON'S LECTURES ON THE PRACTICE OF MEDICINE.

These excellent Lectures are about to be published in London, in two volumes.

### ARAN ON THE HEART.

Messrs. Barrington & Haswell have just published a handsome edition of this excellent manual, in duodecimo, bound in cloth, to match Walshe on the Physical Diagnosis of the Lungs. The student of medicine will find it an excellent guide by the bedside, which will assist him materially in the study and comprehension of a difficult class of diseases. The present portable shape is an additional recommendation.

### ELLIOTSON'S PRACTICE OF MEDICINE.

Our notice of this excellent work, just published by Messrs. Carey and Hart, we have been obliged to postpone till our next number.

### HYDROPATHY.

Dr. Hastings, of Worcester, has detailed an instance of the death-doings of the satellites of Pressnitz, in an old gentleman afflicted with gout, who was credulous enough to undergo the hydropathic treatment at Malvern. A cure was promised in a fortnight, or one hundred pounds to be forfeited in case of failure. The gout, indeed, evacuated the field during the process, and the wily water-doctor saved his conscience by a quibble; for, of course, he was not answerable for the supervention of other and more fatal diseases. The action of the heart soon became seriously affected, and every symptom of hypertrophy of that organ presented itself. The extremities swelled, and a general dropsy came on. In fact, within ten months of the poor fellow's application to the water-quack, he died at Malvern, and at the autopsy his heart was found enlarged to twice its normal size, and the cellular membranes throughout distended with yellow serous fluid. How many of Sir Charles Scudamore's *forty cases* have thus terminated? The after-history of them it was not, of course, his province to disclose!—*Lancet.*

Hydropathy has found its illustrators among the caricaturists, one of whom works in the back shop of Messrs. Fores, in Piccadilly, whence a proof of his labours has been sent to us, under the title of "The sure water-cure;" but as the humour of the lithographist, such as it is, does not legitimately fall under our notice as medical critics, we must pass the work by, extracting, however, one sentence from the preface, which gives to hydropathy a very high authority and most ancient date. "To the idle sneerer at novelties," observes the author, "we proudly point out the fact that"—the water-cure is as old as the flood, the first grand treatment by that remedy having taken place in the time of the venerable Noah.—*Ibid.*

